# Multi-Center Evaluation of the Fully Automated PCR-Based Idylla^™^ KRAS Mutation Assay for Rapid KRAS Mutation Status Determination on Formalin-Fixed Paraffin-Embedded Tissue of Human Colorectal Cancer

**DOI:** 10.1371/journal.pone.0163444

**Published:** 2016-09-29

**Authors:** Jérôme Solassol, Julie Vendrell, Bruno Märkl, Christian Haas, Beatriz Bellosillo, Clara Montagut, Matthew Smith, Brendan O’Sullivan, Nicky D’Haene, Marie Le Mercier, Morten Grauslund, Linea Cecilie Melchior, Emma Burt, Finbarr Cotter, Daniel Stieber, Fernando de Lander Schmitt, Valentina Motta, Calogero Lauricella, Richard Colling, Elizabeth Soilleux, Matteo Fassan, Claudia Mescoli, Christine Collin, Jean-Christophe Pagès, Peter Sillekens

**Affiliations:** 1 Laboratory of Biopathology, Institut du Cancer de Montpellier, Montpellier, France; 2 Institute of Pathology, Klinikum Augsburg, Augsburg, Germany; 3 Pathology Department, Hospital del Mar, IMIM (Hospital del Mar Medical Research Institute), Barcelona, Spain; 4 Oncology Department, Hospital del Mar, IMIM, Barcelona, Spain; 5 Queen Elizabeth Hospital, Birmingham, United Kingdom; 6 Department of Pathology, Hôpital Erasme - Université Libre de Bruxelles, Brussels, Belgium; 7 Department of Pathology, Rigshospitalet Copenhagen, Copenhagen, Denmark; 8 Royal London Hospital, London, United Kingdom; 9 Molecular Genetic Unit, Laboratoire National de Santé, Dudelange, Luxembourg; 10 Department of Pathology, Laboratoire National de Santé, Dudelange, Luxembourg; 11 Molecular Pathology Unit, Department of Laboratory Medicine, Niguarda Cancer Center, ASST - Grande Ospedale Metropolitano Niguarda, Milan, Italy; 12 Department of Cellular Pathology, Oxford University Hospitals NHS Foundation Trust, Oxford, United Kingdom; 13 Department of Medicine (DIMED), Surgical Pathology Unit, University of Padua, Padua, Italy; 14 Platform of Somatic Tumor Molecular Genetics, Centre Hospitalier Régional Universitaire de Tours, Tours, France; 15 Biocartis, Mechelen, Belgium; University of Crete, GREECE

## Abstract

Since the advent of monoclonal antibodies against epidermal growth factor receptor (EGFR) in colorectal cancer therapy, the determination of RAS mutational status is needed for therapeutic decision-making. Most prevalent in colorectal cancer are *KRAS* exon 2 mutations (40% prevalence); lower prevalence is observed for *KRAS* exon 3 and 4 mutations (6%) and *NRAS* exon 2, 3, and 4 mutations (5%). The Idylla^™^ KRAS Mutation Test on the molecular diagnostics Idylla^™^ platform is a simple (<2 minutes hands-on time), highly reliable, and rapid (approximately 2 hours turnaround time) *in vitro diagnostic* sample-to-result solution. This test enables qualitative detection of 21 mutations in codons 12, 13, 59, 61, 117, and 146 of the *KRAS* oncogene being clinically relevant according to the latest clinical guidelines. Here, the performance of the Idylla^™^ KRAS Mutation Assay, for Research Use Only, was assessed on archived formalin-fixed paraffin-embedded (FFPE) tissue sections by comparing its results with the results previously obtained by routine reference approaches for *KRAS* genotyping. In case of discordance, samples were assessed further by additional methods. Among the 374 colorectal cancer FFPE samples tested, the overall concordance between the Idylla^™^ KRAS Mutation Assay and the confirmed reference routine test results was found to be 98.9%. The Idylla^™^ KRAS Mutation Assay enabled detection of 5 additional KRAS-mutated samples not detected previously with reference methods. As conclusion the Idylla^™^ KRAS Mutation Test can be applied as routine tool in any clinical setting, without needing molecular infrastructure or expertise, to guide the personalized treatment of colorectal cancer patients.

## Introduction

The Kirsten rat sarcoma viral oncogene (*KRAS*) belongs to a family of related *RAS* genes, comprising three known human isoforms, i.e., *KRAS*, *NRAS*, and *HRAS* [1, 2]. *KRAS* encodes the KRAS protein, consisting of 188 or 189 amino acids (depending on exon 4 utilization), which is a small membrane-bound GTPase that plays a pivotal role in cell signal transduction. Normally, KRAS exists in an inactive state. Ligand binding of a nearby transmembrane tyrosine kinase receptor, like the epidermal growth factor receptor (EGFR), leads to activation of KRAS, which is directly downstream of this receptor. Once in its active state, KRAS in turn activates a wide variety of downstream effectors, hence influencing cell proliferation and cell survival. Mutated KRAS remains in the active state, leading to a loss of its regulatory function on downstream effectors and eventually to cancer cell survival.

There are several etiological pathways leading to colorectal cancer, the traditional biomarkers being microsatellite instability, the CpG island methylator phenotype, and somatic mutations in *BRAF* and *KRAS* [3–5]. In a recent analysis of randomized controlled trials in metastatic colorectal cancer patients, *RAS* mutation prevalence was found to be 55.9%, with *KRAS* exon 2 mutations being most common (42.6% prevalence), followed by *KRAS* exon 4 (6.2%), *NRAS* exon 3 (4.2%), *KRAS* exon 3 (3.8%), *NRAS* exon 2 (2.9%), and *NRAS* exon 4 (0.3%) mutations [6]. The corresponding *KRAS* single amino acid missense mutations are located at codons 12 or 13 for exon 2, codons 59 or 61 for exon 3, and codons 117 or 146 for exon 4, with G12D (13.1% prevalence), G12V (11.6%), and G13D (8.1%) being most prevalent in colorectal cancer.

Colorectal cancer is the second most common cause of cancer death in Europe, the third most common cause in the US, and the fourth most common cause worldwide [5, 7, 8]. For many years, intravenous 5-fluorouracil/leucovorin or oral capecitabine were the backbone of first-line palliative chemotherapy for colorectal cancer [5, 9]. Since 2000, the addition to 5-fluorouracil/leucovorin of oxaliplatin (FOLFOX) or irinotecan (FOLFIRI), or the combination of capecitabine with oxaliplatin (CAPOX), led to increased response rates and survival. More recently, the advent of targeted therapies including human vascular endothelial growth factor (VEGF) and EGFR monoclonal antibodies, expanded treatment options and further increased treatment response. Thanks to the improvements in treatment and detection, 5-year colorectal cancer survival rates increased significantly from about 50% in the 1970s to about 65% currently [8], while median overall survival of patients with metastatic disease increased from 8–12 months to 21–24 months [10].

Addition of the anti-EGFR monoclonal antibody cetuximab or panitumumab to chemotherapy regimens improves outcome for metastatic colorectal cancer [9]. It was found that presence of mutations in *KRAS* exon 2 (codon 12/13) considerably reduced the efficacy of these EGFR inhibitors [11, 12]. Exon 2 mutations have, therefore, been used as routine biomarkers for predicting lack of response to cetuximab and panitumumab, thereby protecting metastatic colorectal cancer patients from the undesirable side effects and the considerable cost of ineffective therapy. As the presence of exon 2 mutations did not fully explain poor response to anti-EGFR therapy, further analysis identified additional mutations predicting resistance, i.e., mutations in *KRAS* exon 3 (codons 59 and 61) and 4 (codons 117 and 146), and in *NRAS* exon 2 (codons 12 and 13), exon 3 (codons 59 and 61), and exon 4 (codons 117 and 146) [13–15]. Hence, extended *RAS* analysis, beyond *KRAS* exon 2, is necessary to identify patients eligible for EGFR-targeted therapy. This is reflected by the latest clinical practice guidelines of the European Society for Medical Oncology (ESMO) and the National Comprehensive Cancer Network (NCCN) recommending genotyping of tumor tissue (primary or metastatic) for the presence of *KRAS* exon 2 mutations as well as non-exon 2 mutations and *NRAS* mutations [9, 16].

Various laboratory-based or commercial KRAS mutation assays are used in routine practice and the majority of those have been optimized to be compatible with DNA extracted from formalin-fixed paraffin-embedded (FFPE) samples, which represent the most common form of tumor tissue specimens [17]. However, these assays are characterized by differences in the range of mutations covered, sensitivity, labor, cost, level of automation and multiplexing, and in the need for specialized equipment and highly skilled staff. Importantly, it was recently shown that *KRAS* genotyping results were only made available within 15 days for 82%, 51%, and 98% of tested patients in Europe, Latin America, and Asia, respectively [18]. Since a timeframe of 15 working days is undesirable, in particular for the therapeutic management of rapidly progressing metastatic patients, shorter timelines are necessary. In some countries, guidelines even require results to be available within 8 to 10 days [19].

The Idylla^™^ BRAF Mutation Test, CE IVD, on the Idylla^™^ platform (Biocartis, Mechelen, Belgium) was successfully launched for the fast and accurate detection of BRAF V600 mutations in melanoma patients, directly on FFPE samples [20, 21]. More recently, the Idylla^™^ KRAS Mutation Test, CE IVD, which enables detection of 21 clinically relevant KRAS mutations, was launched, and the Idylla^™^ NRAS Mutation Assay was made available for Research Use Only. Unlike conventional methods that use time-consuming FFPE processing steps, Idylla^™^ enables integration of pre-analytical and analytical processes in a single cartridge, thus eliminating the need of manually and time-consuming successive deparaffinization, tissue digestion, DNA extraction procedures, and PCR set-up steps.

Here, the results of a multi-center study testing archived FFPE tumor samples from colorectal cancer patients for their KRAS mutational status with the Idylla^™^ KRAS Mutation Assay (RUO) are reported. To establish test performance, these results were compared to the results previously obtained for these samples using routine reference methods present in the participating laboratories.

## Materials and Methods

### Tissue Sample Collection

Archived clinical FFPE materials of 374 colorectal cancer patients were selected for this study. Samples were obtained from 12 European centers: Klinikum Augsburg (Augsburg, Germany; n = 29), Hospital del Mar (Barcelona, Spain; n = 31), Queen Elizabeth Hospital (Birmingham, UK; n = 32), Hôpital Erasme (Brussels, Belgium; n = 30), Rigshospitalet Copenhagen (Copenhagen, Denmark; n = 29), Royal London Hospital (London, UK; n = 31), Laboratoire National de Santé (Dudelange, Luxembourg; n = 32), Niguarda Cancer Center (Milano, Italy; n = 34), Institut du Cancer de Montpellier (Montpellier, France; n = 32), Oxford University Hospitals NHS Foundation Trust (Oxford, UK; n = 30), Surgical Pathology Unit (Padua, Italy; n = 30), Centre Hospitalier Régional Universitaire de Tours (Tours, France; n = 34).

**Fig 1:**
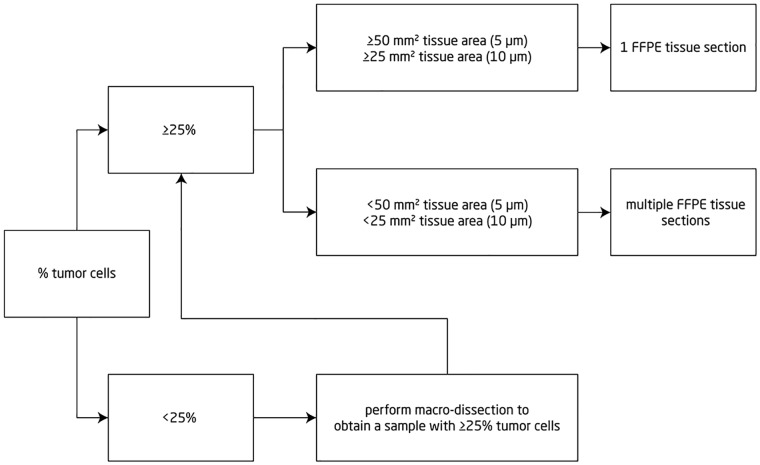
Decision tree of pre-analytical FFPE sample preparation prior to Idylla^™^ KRAS Mutation Assay.

FFPE tissue sections (mostly 1 or 2, up to 12) of 5 μm to 25 μm thick were sampled as close as possible (within the same FFPE block) to the sections used before to generate the reference result (Fig 1). FFPE tissue sections were placed directly into the Idylla^™^ cartridge following the assay instructions. Tumor content and area were determined on hematoxylin-eosin-stained slides by a Pathologist and, if needed, macro-dissection was performed to achieve a tumor cell content of at least 25%.

Artificial FFPE samples (i.e., FFPE Reference Standards from Horizon Discovery, Cambridge, UK) were included as external controls: 5 sites tested KRAS G12D at 5% allelic frequency as well as KRAS G13D at 50% allelic frequency, 2 sites only tested KRAS G12D at 5%, and 1 site only tested KRAS G13D at 50%.

### In-House KRAS Mutation Tests Used as Reference Method

To guide cancer therapy, the KRAS mutational status had been assessed previously on FFPE material using routine reference methods. Depending on each center, different protocols were used.

Sanger sequencing was used as a routine reference method by adopting different protocols and equipment at each site.

Digital droplet PCR was performed on a QX100^™^ system (Bio-Rad Laboratories, Hercules, CA) according to the instructions of the manufacturer.

High-resolution melting (HRM) analysis was performed following three different protocols: (i) fragments from *KRAS* exon 2, 3, and 4 were PCR amplified using a Rotor-Gene 6000 instrument (Qiagen) in combination with the LightCycler 480 High-Resolution Melting Master Reaction Mix (Roche Diagnostics, Meylan, France); (ii) using an automated ABI Prism 7900HT Sequence Detection System (Life Technologies) according to the manufacturer’s recommendations; or (iii) using a LightCycler^®^ 480 II (Roche) using the LightCycler^®^ 480 High Resolution Melting Master Kit (Roche).

Pyrosequencing was performed on a PyroMark Q24 MDx (Qiagen) following the manufacturer’s instructions.

Next-generation-sequencing (NGS) was performed as described [22], using the Ampliseq^™^ Colon and Lung Cancer panel (Life Technologies, Carlsbad, CA) for amplification and sequencing of hotspot mutations in 22 lung and colon cancer relevant genes, and massively parallel sequencing was carried out on an Ion Torrent Personal Genome Machine sequencer (Life Technologies) following the manufacturer’s instructions. Alternatively, the Multiplicom (Niel, Belgium) Somatic 1Mastr v2 Colon Cancer Panel was used for amplification and sequencing of the complete coding regions of the *BRAF*, *KRAS*, and *NRAS* genes, and massively parallel sequencing was carried out on a MiSeq Sequencer (Illumina, San Diego, CA) following the manufacturer’s instructions.

### Commercial Routine KRAS Mutation Tests Used as Reference Method

At several centers to guide cancer therapy, the KRAS mutational status had been assessed previously on FFPE material using commercial routine tests.

The cobas^®^ KRAS Mutation Test (Roche), the Ion Torrent AmpliSeq^™^ Colon and Lung Cancer Research Panel (Thermo Fisher Scientific), therascreen^®^ KRAS Pyro Kit (Qiagen), therascreen^®^ RAS Extension Pyro Kit (Qiagen), the KRAS, BRAF, PIK3CA* Array (Randox Molecular, Crumlin, UK), and the QClamp^™^ KRAS Codon Specific Mutation Detection Kit (Exon 2, 3, 4) (DiaCarta, Hayward, CA), were performed according to the manufacturer’s instructions.

### Idylla^™^ KRAS Mutation Assay

The Idylla^™^ KRAS Mutation Assay enables mutation detection in exons 2, 3, and 4 of the *KRAS* oncogene. This assay consists of five allele-specific multiplex PCR reactions, designed for the specific amplification of *KRAS* gene sequences with a mutation in codons 12, 13, 59, 61, 117, or 146. As such, the test enables detection of 21 KRAS mutations, i.e., seven mutations in exon 2 (codons 12 and 13), nine mutations in exon 3 (codons 59 and 61), and five mutations in exon 4 (codons 117 and 146) of the *KRAS* oncogene. All possible Idylla^™^ KRAS Mutation Assay genetic call values are shown in Table 1. In case of multiple mutations, only the dominantly detected mutation (lowest ΔCq value) is currently reported.

**Table 1:** KRAS mutations detected by the Idylla^™^ KRAS Mutation Test.

Exon	Codon	Mutation	Amino Acid Change	Coding DNA Change	Genetic Call Idylla^™^	LOD allelic frequency Idylla^™^ (%)	Prevalence in colorectal cancer (%)^a^
2	12	G12C	p.Gly12Cys	c.34G>T	G12C	5.0	3.6
		G12R	p.Gly12Arg	c.34G>C	G12R	5.0	0.5
		G12S	p.Gly12Ser	c.34G>A	G12S	5.3	2.5
		G12A	p.Gly12Ala	c.35G>C	G12A	9.1	3.2
		G12D	p.Gly12Asp	c.35G>A	G12D	5.0	13.1
		G12V	p.Gly12Val	c.35G>T	G12V	5.0	11.6
	13	G13D	p.Gly13Asp	c.38G>A	G13D	10.0	8.1
3	59	A59E	p.Ala59Glu	c.176C>A	A59T/E/G	5.0	0.0
		A59G	p.Ala59Gly	c.176C>G	A59T/E/G	5.0	0.0
		A59T	p.Ala59Thr	c.175G>A	A59T/E/G	5.0	0.0
	61	Q61K	p.Gln61Lys	c.181C>A	Q61K	5.0	0.1
		Q61K	p.Gln61Lys	c.180_181TC>AA	Q61K	5.0	0.1
		Q61L	p.Gln61Leu	c.182A>T	Q61L/R	5.0	0.2
		Q61R	p.Gln61Arg	c.182A>G	Q61L/R	5.0	0.3
		Q61H	p.Gln61His	c.183A>C	Q61H	5.0	1.6
		Q61H	p.Gln61His	c.183A>T	Q61H	5.0	1.6
4	117	K117N	p.Lys117Asn	c.351A>C	K117N	5.0	0.5
		K117N	p.Lys117Asn	c.351A>T	K117N	5.0	0.5
	146	A146P	p.Ala146Pro	c.436G>C	A146P/T/V	16.2	0.1
		A146T	p.Ala146Thr	c.436G>A	A146P/T/V	16.2	2.2
		A146V	p.Ala146Val	c.437C>T	A146P/T/V	16.2	0.5

^a^ Individual mutations are shown as a percentage of the total number of mutations [6].

FFPE tissue sections were placed directly into the cartridge of the fully automated Idylla^™^ platform (Biocartis, Mechelen, Belgium) following the manufacturer’s instructions, without requiring prior manual deparaffinization or FFPE pre-processing. Briefly, after insertion of the FFPE tissue section into the cartridge and insertion of the cartridge into the Idylla^™^ instrument, a combination of reagents, enzymes, heat, and high intensity focused ultrasound (HIFU) induces deparaffinization, disruption of the tissue, and lysis of the cells. The nucleic acids are liberated for subsequent real-time PCR amplification. Allele-specific primers and fluorescent probes are present in a stable formulation. A sample processing control, involving the simultaneous amplification of a conserved fragment in the intron 4 / exon 5 junctional region of the *KRAS* gene, was performed in each of the five multiplex PCR reactions to check for adequate execution of the complete sample-to-result process and as a measure for the amount of amplifiable DNA in the sample.

For every valid PCR curve, the Idylla^™^ software calculates a quantification cycle (Cq) value. If the difference between the measured Cq for a KRAS mutant PCR signal and the KRAS wild-type Cq value, i.e. the ΔCq value, is within a validated range, the sample is characterized as KRAS mutation positive, and the specific mutation or mutation group is indicated. Samples having a valid KRAS wild-type signal but a ΔCq value outside the validated range are reported as being KRAS mutation negative (wild-type). Results expressed as invalid are due to the presence of inhibitors in the sample, insufficient amplifiable DNA present in the sample, incorrect placement of the sample in the cartridge, or a sample volume that is out of range. In addition, incorrectly stored cartridges, use of cartridges that exceeded their in-use period after removal from the pouch, or cartridge malfunctioning can cause invalid results.

The results obtained with the Idylla^™^ KRAS Mutation Assay on archival material were not used for diagnostic purposes of any kind.

### Analytical Sensitivity and Specificity of Idylla^™^ KRAS Mutation Test

Both analytical sensitivity and specificity have been established for the Idylla^™^ KRAS Mutation Test (CE IVD). Idylla^™^ KRAS Mutation Test (CE IVD) and Idylla^™^ KRAS Mutation Assay (RUO) both consist of the same design and decision tree.

The test enables identifying the presence of a KRAS mutation with a limit of detection (LOD) of ≤5% allelic frequency in a standard Horizon FFPE tissue section for the vast majority of KRAS mutations, while G12A, G13D, and A146P/T/V show LOD values of 9%, 10%, and 16%, respectively (Table 1).

The LOD is defined as the lowest KRAS mutation copy number consistently detected in ≥95% of cases (with 95% confidence), at an allelic frequency of 5%. For KRAS mutations G12A, G12C, G12D, G12R, G12S, G12V, G13D, Q61H, Q61L, and A146T, dilution series were prepared by blending liquefied KRAS mutant FFPE material with liquefied KRAS wild-type FFPE material to the desired allelic frequency, and for mutations Q61K (c.180_181TC>AA), K117N (c.351A>T), and A59E (c.176C>A), synthetic target DNA was spiked to the desired allelic frequency into liquefied KRAS wild-type FFPE material. Two-fold dilution series were prepared in 12-fold and tested with cartridges from two Idylla^™^ KRAS Mutation Test lots. Mutation call rates were used to determine the LOD by logistic regression, and the corresponding tissue area equivalent of an artificial FFPE specimen with the allelic frequency as indicated (Table 1) was deduced from these copy numbers. LOD values were verified by testing 20 replicates at or below the upper limit of the 95% confidence interval.

*In silico* analysis of the human genome sequence did not identify reactivity for any of the oligonucleotide primers outside the *KRAS* gene that could possibly result in non-specific amplicon formation and/or detection, thereby excluding cross-reactivity of Idylla^™^ KRAS Mutation Test primers with sequence homologues in the *NRAS* gene or the *KRASP1* pseudogene. Screening of single point mutations (SNPs) reported for the human *KRAS* gene by *in silico* analysis, did not reveal any mutations outside the codons targeted by the Idylla^™^ KRAS Mutation Test that could lead to false-positive results for KRAS. Known variants for the KRAS mutations targeted by the Idylla^™^ KRAS Mutation Test were identified in this *in silico* SNP analysis to be detected and correctly reported by the assay. The only exceptions that will not be detected are two rarely occurring variants for G12V and Q61R, respectively. Of the known mutations within the codons covered by the Idylla^™^ KRAS Mutation Test but for which the primers were not primarily designed, mutation G12W will be detected and reported as G12C, and mutations G13N and G13E will result in a G13D call.

### Statistical Analysis

For method correlation, the one-sided 95% confidence intervals were calculated using the Wilson method. Percentage agreement was calculated for the Idylla^™^ KRAS Mutation Assay against routine reference methods. When comparing two methods, overall percent agreement was calculated from the number of specimens tested positive and negative by both methods, and the total number of specimens. Positive percent agreement was calculated from the number of specimens tested positive by both methods, and the total numbers of specimens tested positive for the reference method. Negative percent agreement was calculated from the number of specimens tested negative by both methods, and the total numbers of specimens tested negative for the reference method.

## Results

### Inter-Laboratory Reproducibility Idylla^™^ KRAS Mutation Assay

Artificial FFPE samples (i.e., FFPE Reference Standards from Horizon Discovery) were included as external controls: 5 sites tested KRAS G12D at 5% allelic frequency and KRAS G13D at 50% allelic frequency, 2 sites only tested KRAS G12D at 5%, and 1 site only tested KRAS G13D at 50%.

The assay identified each of these mutations correctly (testing 1 replicate per mutation per site). Hence, inter-laboratory reproducibility was 100% (based on 6 out of 6 G13D, and 7 out of 7 G12D).

### Idylla^™^ KRAS Mutation Assay

Using the Idylla^™^ KRAS Mutation Assay, the KRAS mutational status of 374 archived clinical colorectal cancer FFPE samples was tested at 12 centers. The Idylla^™^ results were compared with the original assessments made by routine reference methods (Tables 2 and 3, and S1 Table).

**Table 2:** Comparison between results of the Idylla^™^ KRAS Mutation Assay and of routine reference methods.

		**Routine reference methods**^a^																
		**G12A**	**G12C**	**G12D**	**G12R**	**G12S**	**G12V**	**G13D**	**A59G/T/E**	**Q61L/R**	**Q61H**	**Q61K**	**K117N**	**A146P/T/V**	**WT**	**ND**	**Rare mutations (<1%)**^b^	**Total**
**Idylla**^**™**^	**G12A**	**13**	0	0	0	0	0	0	0	0	0	0	0	0	0	0	0	13
	**G12C**	0	**17**	0	0	0	0	0	0	0	0	0	0	0	1	0	0	18
	**G12D**	0	0	**56**	0	0	0	0	0	0	0	0	0	0	1	0	0	57
	**G12R**	0	0	0	**5**	0	0	0	0	0	0	0	0	0	0	0	0	5
	**G12S**	0	0	0	0	**9**	0	0	0	0	0	0	0	0	0	1^d^	0	10
	**G12V**	0	0	2	0	0	**36**	0	0	0	0	0	0	0	1	0	0	39
	**G13D**	0	0	0	0	0	0	**38**	0	0	0	0	0	0	0	0	0	38
	**A59G/T/E**	0	0	0	0	0	0	0	**5**	0	0	0	0	0	0	0	0	5
	**Q61L/R**	0	0	0	0	0	0	0	0	**6**	0	0	0	0	2	0	0	8
	**Q61H**	0	0	0	0	0	1	0	0	0	**6**	0	0	0	4	0	0	11
	**Q61K**	0	0	0	0	0	0	0	0	0	0	**2**	0	0	0	0	0	2
	**K117N**	0	0	0	0	0	0	0	0	0	0	0	**4**	0	0	0	0	4
	**A146P/T/V**	0	0	0	0	0	0	0	0	0	0	0	0	**18**	1	2^c^	0	21
	**WT**	1^e^	0	1	0	0	0	2^e^	0	0	0	0	0	1	**129**	0	0	134
	**ND**	0	0	0	0	0	1^d^	1^d^	0	0	0	0	0	0	0	0	7^e^	9
	**Total**	14	17	59	5	9	38	41	5	6	6	2	4	19	139	3	7	374

WT, wild type; ND, not detectable (including invalid result).

^a^ Different reference methods were used: cobas^®^ KRAS Mutation Test (Roche), Ion Torrent AmpliSeq^™^ Colon and Lung Cancer Research Panel (Life Technologies), therascreen^®^ KRAS Pyro^®^ Kit (Qiagen), therascreen^®^ RAS Extension Pyro Kit (Qiagen), HRM screening and pyrosequencing, Sanger sequencing, HRM screening and Sanger sequencing; for the analysis, when Idylla^™^ identified a specific mutation in codon 12, 13 or 61, and the cobas^®^ KRAS Mutation Test (Roche) reported a “codon 12/13” or “codon 61” result), both results were considered identical.

^b^ Rare mutations: G12F, G13C, G13R, double mutation.

^c^ cobas^®^ KRAS Mutation Test (Roche); detects only mutations in codon 12/13 and in codon 61.

^d^ Invalid result.

^e^ Codon 12/13 mutations detected by the cobas^®^ KRAS Mutation Test (Roche) were further analyzed with additional reference methods (see below) to determine the exact nature of the mutation found.

**Table 3:** Comparison between results of the Idylla^™^ KRAS Mutation Assay and of routine reference methods (codon level).

		**Pyrosequencing**^a^								
		**WT**	**c12**	**c13**	**c59**	**c61**	**c117**	**c146**	**Invalid**	**Total**
**Idylla**^**™**^	**WT**	***51***	2^b^	4	0	0	0	0	0	57
	**c12**	1	***35***	0	0	0	0	0	1	37
	**c13**	0	0	***11***	0	0	0	0	0	11
	**c59**	0	0	0	***1***	0	0	0	0	1
	**c61**	0	0	0	0	***8***	0	0	0	8
	**c117**	0	0	0	0	0	***2***	0	0	2
	**c146**	1	0	0	0	0	0	***10***	0	11
	**Invalid**	0	1	1	0	0	0	0	***0***	2
	**Total**	53	38	16	1	8	2	10	1	129
		**cobas**^c^								
		**WT**	**c12/c13**		**c59**	**c61**	**c117**	**c146**	**Invalid**	**Total**
**Idylla**^**™**^	**WT**	***44***	4		0	0	0	0	0	48
	**c12**	2	***47***		0	0	0	0	0	49
	**c13**	0	***14***		0	0	0	0	0	14
	**c59**	0	0		***0***	0	0	0	0	0
	**c61**	1	0		0	***4***	0	0	0	5
	**c117**	0	0		0	0	***0***	0	0	0
	**c146**	3	0		0	0	0	***0***	0	3
	**Invalid**	0	0		0	0	0	0	***0***	0
	**Total**	50	65		0	4	0	0	0	119
		**NGS**^d^								
		**WT**	**c12**	**c13**	**c59**	**c61**	**c117**	**c146**	**Invalid**	**Total**
**Idylla**^**™**^	**WT**	***28***	0	0	0	0	0	0	0	28
	**c12**	0	***13***	0	0	0	0	0	0	13
	**c13**	0	0	***4***	0	0	0	0	0	4
	**c59**	0	0	0	***2***	0	0	0	0	2
	**c61**	0	0	0	0	***1***	0	0	0	1
	**c117**	0	0	0	0	0	***1***	0	0	1
	**c146**	0	0	0	0	0	0	***3***	0	3
	**Invalid**	0	0	0	0	0	0	0	***0***	0
	**Total**	28	13	4	2	1	1	3	0	52
		**Overall**^e^								
		**WT**	**c12**	**c13**	**c59**	**c61**	**c117**	**c146**	**Invalid**	**Total**
**Idylla**^**™**^	**WT**	***129***	4^b^	7	0	0	0	1	0	141
	**c12**	3	***138***	0	0	0	0	0	1	142
	**c13**	0	0	***38***	0	0	0	0	0	38
	**c59**	0	0	0	***5***	0	0	0	0	5
	**c61**	6	1	0	0	***14***	0	0	0	21
	**c117**	0	0	0	0	0	***4***	0	0	4
	**c146**	3	0	0	0	0	0	***18***	0	21
	**Invalid**	0	1	1	0	0	0	0	***0***	2
	**Total**	141	144	46	5	14	4	19	1	374

^a^ therascreen^®^ KRAS Pyro Kit (Qiagen), therascreen^®^ RAS Extension Pyro Kit (Qiagen), in-house pyrosequencing.

^b^ One of the samples contains double mutation G12A+G13R.

^c^ cobas^®^ KRAS Mutation Test (Roche); detects mutations in codon 12/13 and in codon 61.

^d^ Ion Torrent AmpliSeq^™^ Colon and Lung Cancer Research Panel (Life Technologies).

^e^ Including results from other reference methods. For this analysis, codon 12/13 mutations detected by the cobas^®^ KRAS Mutation Test (Roche) were categorized as codon 12 or codon 13 mutation, respectively, taking into account the mutation detected by the Idylla^™^ KRAS Mutation Assay, or by further analysis (see below).

Several routine reference methods were used: cobas^®^ KRAS Mutation Test (Roche; n = 119), Ion Torrent AmpliSeq^™^ Colon and Lung Cancer Research Panel (Life Technologies, n = 52), therascreen^®^ KRAS Pyro Kit (Qiagen, n = 85), therascreen^®^ RAS Extension Pyro Kit (Qiagen, n = 57), HRM screening (combined with the previous 2 therascreen^®^ kits, n = 34), HRM screening and pyrosequencing (n = 32), HRM screening and Sanger (n = 34), Sanger sequencing (as only method n = 59). Macro-dissection was performed in 141 cases to enrich tumor area, in order to maximize sensitivity.

Of the 374 FFPE samples analyzed by the Idylla^™^ KRAS Mutation Assay, 231 tested positive for a mutation in *KRAS* codon 12, 13, 59, 61, 117, or 146 (Table 4A): 13 samples with mutation G12A, 18 with G12C, 57 with G12D, 5 with G12R, 10 with G12S, 39 with G12V, 38 with G13D, 5 with A59T/E/G, 2 with Q61K, 8 with Q61L/R, 11 with Q61H, 4 with K117N, and 21 with A146P/T/V. In 141 cases no mutation was found, and an “invalid” call was reported in 2 cases with the Idylla^™^ KRAS Mutation Assay and in 1 case with the therascreen^®^ KRAS Pyro Kit (Qiagen) (Table 4A). Overall, in this first assessment, agreement between the Idylla^™^ results and the results of the routine reference methods was observed in 347 cases. These 347 cases include 3 cases in which a different KRAS mutation was identified by the Idylla^™^ KRAS Mutation Assay as compared to the routine reference method (Table 2). However, as treatment decisions are driven by the mere absence or presence of a mutation in one of the relevant codons and are not influenced by the exact nature of such a mutation, these 3 Idylla^™^ results were classified as being concordant.

**Table 4:** Comparison between results of the Idylla^™^ KRAS Mutation Assay and (A) results of routine reference methods, or (B) results of routine reference methods including further analysis.

		**(A)**				**(B)**		
		**Routine reference methods**^a^				**Routine reference methods including further analysis**^a^		
		**Mutation**	**No mutation**	**Invalid**	**Total**	**Mutation**	**No mutation**	**Total**
**Idylla**^**™**^	**Mutation**	218	12	1	231	224	1	225
	**No mutation**	12	129	0	141	3	134	137
	**Invalid**	2	0	0	2	NA	NA	NA
	**Total**	232	141	1	374	227	135	362
**Idylla**^™^ **Performance**	**Positive agreement**^b^	94.78% (95% CI: 91.81% - 100%)				98.68% (95% CI: 96.74% - 100%)		
	**Negative agreement**^b^	91.49% (95% CI: 86.8% - 100%)				99.25% (95% CI: 96.75% - 100%)		
	**Overall agreement**^b^	93.53% (95% CI: 91.1% - 100%)				98.89% (95% CI: 97.56% - 100%)		

NA, not applicable; 95% CI, 95% confidence interval.

^a^ Different reference methods were used: cobas^®^ KRAS Mutation Test (Roche), Ion Torrent AmpliSeq^™^ Colon and Lung Cancer Research Panel (Life Technologies), therascreen^®^ KRAS Pyro^®^ Kit (Qiagen), therascreen^®^ RAS Extension Pyro Kit (Qiagen), HRM screening and pyrosequencing, Sanger sequencing, and HRM screening and Sanger sequencing; and for further analysis KRAS BRAF PIK3CA* Array (Randox Molecular), QClamp^™^ KRAS Codon Specific Mutation Detection Kit (Exon 2, 3, 4) (DiaCarta), ddPCR, and Illumina NGS as well.

^b^ Positive, negative, and overall agreement values were calculated not taking the invalids into account.

In 12 cases, no KRAS mutation was detected by the Idylla^™^ KRAS Mutation Assay while the reference method had identified a mutation. In another 12 cases, the Idylla^™^ KRAS Mutation Assay identified a mutation that had not been detected by the routine reference method. These 24 discordant samples were further analyzed (Table 5).

**Table 5:** Discordant results between the Idylla^™^ KRAS Mutation Assay and routine reference methods.

Sample #	Tissue type	FFPE tissue section (μm)	Number of FFPE tissue sections	Tumor cells (%)	Tumor area mm^2^	Macro-dissection	Idylla^™^	Routine reference method	Further analysis	Conclusion based on further analysis
**Discordant results**										
Au_06	colorectal	10	2^a^	>50	30	no	no mutation	A146T^d^	A146T 29.7%^k^; A146T^l^	C*
Bi_04	colon	6	1	20	398	no	G12C	no mutation^h^^,^^e^	no mutation^l^^,^^n^	C*
Du_26	colorectal	10	1^b^	50	63	no	no mutation	codon 12/13^e^	G12A 7.3%^m^; G12A 4.7%^k^; G12A^l^	C*
Au_18	metastasis	5	2	>50	15	yes	no mutation	G12D^d^	G12D 0.87%^k^	D
Lo_23	colon	5	1	65	500	no	no mutation	codon 12/13^e^	G13D 0.45%^k^	D
Mo_14	colorectal	10	10	60	65	yes	no mutation	G13D^f^	G13D 0.11%^k^	D
Mo_32	colorectal	10	10	10	30	yes	no mutation	G12-G13>AR^f^	no mutation^k^	C
Mi_13	colon	10	1	50	225	no	G12D	no mutation^g^	G12D 5.73%^k^	C
Ox_13	colorectal	5	1	>50	50–600	yes	G12V	no mutation^e^	G12V 1.8%^k^	C
Pa_12	liver	10	1	40	70	no	Q61R/L	no mutation^d^	Q61R 0.11%^k^	C
Bi_15	liver	6	1	10	340	no	A146P/T/V	no mutation^h^^,^^e^	A146T^n^	C
Mi_03	lymph node	5	2	60	70	no	Q61H^c^	no mutation^g^	no mutation^k^^,^^l^	C*
Mi_07	colon	10	1	60	125	no	Q61H^c^	no mutation^g^	no mutation^k^^,^^l^	C*
Mi_31	colon	10	1	80	325	no	Q61H^c^	no mutation^g^	no mutation^k^^,^^l^	C*
Mi_10	liver	5	2	60	50	no	Q61H^c^	no mutation^g^	ND	X
Ox_28	colorectal	5	1	>50	50–600	yes	Q61L/R	no mutation^e^	no mutation^k^	D
Discordant results by design (i.e. mutation not detectable by method used)										
Bi_01	liver	6	3	70	12	no	no mutation	G13C^i^^,^^e^	ND	X
Bi_07	rectum	6	4	30	60	no	no mutation	G13C^i^	ND	X
Co_13	colorectal	5	2	30	50	no	no mutation	codon 12/13^e^	G13C^o^	X
Mo_31	colorectal	10	10	50	140	yes	no mutation	G12F^f^	ND	X
Pa_28	colorectal	10	1	35	94	no	no mutation	G13R^d^	ND	X
To_19	colorectal	3	1	60	91	yes	no mutation	G13C^j^	ND	X
Du_05	colorectal	10	1	40	100	no	A146P/T/V	no mutation^e^	ND	X
Lo_12	colon	5	1	90	250	no	A146P/T/V	no mutation^e^	A146T 25.5%^k^	X

C, concordant; C*, concordant after Idylla^™^ KRAS Mutation Assay retesting; D, discordant; X, excluded from dataset; ND, not determined (in most cases no tissue block left for further analysis).

^a^ Retested three times with the Idylla^™^ KRAS Mutation Assay using one tenth of the original sample.

^b^ Three times more input is used when retested with the Idylla^™^ KRAS Mutation Assay.

^c^ Probably contamination issue in the laboratory.

^d^ Sanger sequencing.

^e^ cobas^®^ KRAS Mutation Test (Roche).

^f^ HRM + pyrosequencing + Sanger sequencing.

^g^ HRM + Sanger sequencing.

^h^ therascreen^®^ KRAS Pyro Kit (Qiagen) + therascreen^®^ RAS Extension Pyro Kit (Qiagen).

^i^ therascreen^®^ KRAS Pyro Kit (Qiagen)

^j^ HRM + therascreen^®^ KRAS Pyro Kit (Qiagen).

^k^ ddPCR.

^l^ Idylla^™^ KRAS Mutation Assay retesting.

^m^ Illumina NGS.

^n^ KRAS, BRAF, PIK3CA* Array (Randox Molecular) and QClamp^™^ KRAS Codon Specific Mutation Detection Kit (Exon 2, 3, 4) (DiaCarta).

^o^ Ion Torrent AmpliSeq^™^ Colon and Lung Cancer Research Panel (Life Technologies).

### Discordant Results: Mutation Detected by Routine Reference Method and Not By Idylla^™^ KRAS Mutation Assay

In FFPE material of 12 colorectal cancer patients, a mutation in the *KRAS* oncogene previously detected by a routine reference method was not detected when reanalyzing the clinical archival material with the Idylla^™^ KRAS Mutation Assay (Table 5).

As the Idylla^™^ KRAS Mutation Assay is not intended to identify mutations G12F, G13C, or G13R, the “no mutation” call for the 6 samples (Bi_01, Bi_07, Co_13, Mo_31, Pa_28, and To_19) harboring one of these mutations was expected. Hence, no further analysis was performed on 5 of these samples. For sample Co_13, the presence of a mutation in codon 13 but with identity G13C was confirmed by NGS.

For sample Au_06, the presence of the A146T mutation detected earlier with Sanger sequencing was confirmed by ddPCR, which detected A146T with 29.7% allelic frequency. Therefore, slices from the same FFPE block were retested three times with the Idylla^™^ KRAS Mutation Assay using one tenth of the original sample, and each time an A146P/T/V call was obtained. The exact reason why the Idylla^™^ KRAS Mutation Assay originally missed this mutation was not further investigated.

ddPCR confirmed the presence of mutation G12D in sample Au_18, although at an allelic frequency (0.87%) below the LOD of the Idylla^™^ KRAS Mutation Assay for this mutation, which may have been the reason for not detecting it with Idylla^™^. The allelic frequency might have decreased when cutting further into the block.

In sample Lo_23, ddPCR confirmed the presence of G13D at low allelic frequency (0.45%), which is below the LOD of the Idylla^™^ KRAS Mutation Assay for this mutation (10%).

Mutation G12A in sample Du_26 was not detected by the Idylla^™^ KRAS Mutation Assay, while its presence was confirmed by both Illumina NGS (7.3% allelic frequency) and ddPCR (4.7% allelic frequency). Because the allelic frequency value of G12A was around the LOD of the Idylla^™^ KRAS Mutation Assay for this mutation (9%), insufficient sample input might be the cause for not detecting the mutation at this level of allelic frequency. Retesting FFPE slices of this sample with the Idylla^™^ KRAS Mutation Assay, while using three times more input material, indeed resulted in successful detection of G12A.

In sample Mo_14, ddPCR confirmed the presence of mutation G13D, although at a low allelic frequency (0.11%). Hence, the presence of this mutation in the sample was well below the Idylla^™^ LOD for this mutation. Additionally, in this sample mutation V14I was identified by HRM and pyrosequencing; the presence of this double mutation (G13D+V14I) in close vicinity of each other most likely has affected detection by the Idylla^™^ KRAS Mutation Assay as well.

The Idylla^™^ KRAS Mutation Assay normally detects mutation G12A but not G13R, the presence of both mutations in close vicinity of each other in sample Mo_32 might have hindered the detection of G12A. Moreover ddPCR did not confirm the presence of mutation G12A in sample Mo_32, but this ddPCR result might also have been affected by the presence of the double mutation.

Overall, in 4 samples (Au_18, Lo_23, Mo_14, and Mo_32), a mutation detected by a routine reference method was not detected by the Idylla^™^ KRAS Mutation Assay. Of note, in three of these cases, the mutation was present at an allelic frequency below the Idylla^™^ LOD, possibly due to decreasing allelic frequencies when cutting further into the block.

### Discordant Results: Mutation Detected by Idylla^™^ KRAS Mutation Assay and Not By Routine Reference Method

In 12 of the archival clinical FFPE samples tested in this study, the Idylla^™^ KRAS Mutation Assay identified mutations that were not identified by routine reference methods before (Table 5).

ddPCR analysis of the samples confirmed the mutations found by the Idylla^™^ KRAS Mutation Assay in 4 samples (Lo_12, Mi_13, Ox_13, and Pa_12). Although A146T was present in an allelic frequency of 25.5% in sample Lo_12, the cobas^®^ KRAS Mutation Test (Roche) was not able to detect this mutation, which is expected as this test is not designed for the identification of mutations in codons 59, 117, and 146 of the *KRAS* oncogene.

For sample Bi_15, the mutation identified by the Idylla^™^ KRAS Mutation Assay was confirmed by the KRAS, BRAF, PIK3CA* Array (Randox Molecular) and by the QClamp^™^ KRAS Codon Specific Mutation Detection Kit (Exon 2, 3, 4) (DiaCarta).

In 3 samples (Mi_03, Mi_07, and Mi_31), ddPCR did not detect the Q61H mutation identified by the Idylla^™^ KRAS Mutation Assay. Moreover, a repeat test of these samples did not identify this mutation anymore either. Hence, the original discordance between the Idylla^™^ KRAS Mutation Assay and the routine reference method might have been due to a contamination issue in the laboratory. The results for sample Mi_10 might have been affected by the same issue, however there was no material left for further analysis and possible confirmation of this hypothesis.

For sample Du_05 no further analysis was performed as the “no mutation” call of the cobas^®^ KRAS Mutation Test (Roche) for *KRAS* exon 4 mutations was expected. The Idylla^™^ KRAS Mutation Assay identified mutation A146P/T/V.

Concerning sample Bi_04, the Idylla^™^ KRAS Mutation Assay initially found mutation G12C while the therascreen^®^ KRAS Pyro Kit (Qiagen) and therascreen^®^ RAS Extension Pyro Kit (Qiagen) did not find this mutation. The “no mutation” finding was confirmed by the KRAS, BRAF, PIK3CA* Array (Randox Molecular) and QClamp^™^ KRAS Codon Specific Mutation Detection Kit (Exon 2, 3, 4) (DiaCarta). Retesting with the Idylla^™^ KRAS Mutation Assay did no longer find the G12C mutation.

In sample Ox_28, the initial cobas^®^ KRAS Mutation Test result was confirmed by ddPCR.

In total, in 5 samples (Bi_15, Lo_12, Mi_13, Ox_13, and Pa_12), the Idylla^™^ KRAS Mutation Assay was able to identify a mutation that was not identified by the first-line routine reference method but that was confirmed during further analysis.

### Performance of the Idylla^™^ KRAS Mutation Assay

Further analysis enabled the identification of 8 samples (Bi_15, Mi_03, Mi_07, Mi_13, Mi_31, Mo_32, Ox_13, and Pa_12) for which the Idylla^™^ KRAS Mutation Assay result was confirmed by an independent reference method, and in 3 samples (Au_06, Bi_04, and Du_26) Idylla^™^ KRAS Mutation Assay retesting results were in agreement with the reference method result. Therefore, the number of concordant samples was increased from 347 to 358 upon discordance analysis.

The Idylla^™^ KRAS Mutation Assay is not intended to identify the rare mutations G12F, G13C, or G13R (<0.5% prevalence in colorectal cancer), which were previously detected in 6 archival FFPE samples by routine reference methods. Despite the correct “no mutation detected” Idylla^™^ call, these 6 samples (Bi_01, Bi_07, Co_13, Mo_31, Pa_28, and To_19) were excluded from the dataset. Likewise, samples Du_05 and Lo_12 were excluded from the dataset as the reference method used is not designed to detect the *KRAS* exon 4 mutation identified by the Idylla^™^ KRAS Mutation Assay. Sample Mi_10 is removed from the dataset as there was no material left to confirm the mutation found by the Idylla^™^ KRAS Mutation Assay, while a contamination issue was suspected. Also the samples with invalid results for the Idylla^™^ KRAS Mutation Assay (2 samples) or the reference method (1 sample) were removed from the dataset. As a result, the total number of samples was reduced to 362.

Taking into account the additional concordant samples and the decreased number of samples in the dataset, the results obtained by the Idylla^™^ KRAS Mutation Assay were in agreement with the confirmed reference method results in 358 out of 362 samples, resulting in an overall concordance of 98.89% (95% confidence interval: 97.56% - 100%) (Table 4B). The negative percent agreement was 99.25% (95% confidence interval: 96.75% - 100%) and positive percent agreement was 98.68% (95% confidence interval: 97.74% - 100%).

## Discussion

The innovative Idylla^™^ KRAS Mutation Test, performed on the molecular diagnostic Idylla^™^ platform (Biocartis, Mechelen, Belgium), enables the qualitative detection in human colorectal cancer FFPE material of 21 KRAS mutations being clinically relevant according to the latest CAP/AMP/ASCO, ESMO, and NCCN guidelines [23]. These mutations encompass codons 12, 13, 59, 61, 117, and 146 of the *KRAS* oncogene.

Here, an analysis comparing the Idylla^™^ KRAS Mutation Assay (RUO) results with the original assessments made by routine reference methods for determining the KRAS mutational status on FFPE samples is reported. Twelve clinical centers contributed to this study, and a total of 374 archival clinical colorectal cancer FFPE samples were tested. The samples were specifically selected for this study; hence the mutational rates in the current sample set are not representative for the incidence of KRAS mutations in the general colorectal cancer patient population. The results showed an overall presence of KRAS mutations in the samples of 62.0% when measured with routine reference methods and of 61.8% measured with the Idylla^™^ KRAS Mutation Assay, demonstrating that the sensitivity of the Idylla^™^ KRAS Mutation Assay is comparable to the sensitivity currently used routine reference methods. The overall concordance between the Idylla^™^ KRAS Mutation Assay and the confirmed reference routine test results for colorectal cancer samples was found to be 98.9%, with a negative percent agreement of 99.25% and a positive percent agreement of 98.68%.

The initial results showed discordances between results of the Idylla^™^ KRAS Mutation Assay and of the original routine reference method for 24 of the 374 samples. In 12 of these 24 cases, the Idylla^™^ KRAS Mutation Assay did not identify the KRAS mutation that was previously detected by the reference method, while in another 12 cases the Idylla^™^ KRAS Mutation Assay identified a mutation not detected by the routine reference method. These 24 discordant samples were analyzed further to establish the overall concordance. Compared to pyrosequencing (including in-house methods, and the therascreen^®^ KRAS Pyro Kit and RAS Extension Pyro Kit [Qiagen]), the cobas^®^ KRAS Mutation Test (Roche), and NGS (Ion Torrent AmpliSeq^™^ Colon and Lung Cancer Research Panel [Life Technologies]), the observed concordances varied between 91.5% and 100% for these initial results.

Several reasons were found why the Idylla^™^ KRAS Mutation Assay did not identify KRAS mutations detected earlier by routine reference methods. Six of the 12 specimens displayed G13C, G12F, or G13R *KRAS* exon 2 mutations, which are not included in the Idylla^™^ KRAS Mutation Assay because they are rarely observed (<0.5%) in the large colorectal cancer cohorts published [24–26]. Four other samples exhibited G12A, G12D, or G13D mutations according to the reference results, but the observed allelic frequencies were below the LOD values of the Idylla^™^ KRAS Mutation Assay (i.e., 9% for G12A, 5% for G12D, and 10% for G13D; Table 1); in one of these samples, retesting with three times more input material indeed detected the mutation. A decrease in allelic frequency when cutting further into the block might be an issue in particular when using archival material. In one sample, the presence of an additional mutation may have hindered the detection of the G12A mutation by the Idylla^™^ KRAS Mutation Assay; interestingly, ddPCR could not confirm the presence of mutation G12A in this sample either, demonstrating that the presence of a double mutation located in two consecutive codons can affect allele-specific-based techniques. In addition, co-mutations for G12D and G13D have exceptionally been described in colorectal cancer.

The other way around, the Idylla^™^ KRAS Mutation Assay identified mutations that were not identified by routine reference methods in 12 archival clinical FFPE samples. Three of these cases exhibited mutations with too low allelic mutation frequencies to be detected by the used reference method (HRM, Sanger sequencing, or cobas^®^ KRAS Mutation Test), and two other cases displayed mutations not intended to be detected by the routine approach (i.e., *KRAS* exon 4 mutations by the cobas^®^ KRAS Mutation Test). When considering all KRAS mutations, there was a strong agreement in detection rates between the Idylla^™^ KRAS Mutation Assay and the routine reference methods. Overall, in 5 archival clinical samples, the Idylla^™^ KRAS Mutation Assay detected KRAS mutations (confirmed by further analysis) which were not detected by the routine reference methods previously used to guide therapy.

Of note, the Idylla^™^ KRAS Mutation Assay identified different KRAS mutations in three cases as compared to routine reference methods, but since treatment decisions are driven by the presence of a mutation in the relevant codons and not by its exact nature, these results were considered concordant.

According to manufacturer’s instructions, the minimal tumor cell percentage in samples for the Idylla^™^ KRAS Mutation Assay should be at least 25% to guarantee reliable results. In case this lower limit is not met, macro-dissection should be performed in order to enrich tumor area (and thus tumor cells) and to maximize sensitivity. In the current study, macro-dissection was performed in 141 cases.

It is now well established that the frequency of detected KRAS mutations in colorectal cancer is influenced by the analytical sensitivity of the method applied for their detection. The sensitivity of the Idylla^™^ KRAS Mutation Assay is comparable to most of the routine approaches used in laboratories such as competitive allele-specific Taqman, amplification refractory mutation system-PCR, HRM, and pyrosequencing. These reference methods require process steps including deparaffinization, DNA extraction, PCR amplification, and mutation detection, which does not allow on demand testing and therefore leads to batch testing. Hence turnaround times are more than a day, and even several days in the case of NGS. In addition, they are labor-intensive, and require a specialized staff and complex infrastructure. The Idylla^™^ KRAS Mutation Assay is a simple and fast procedure that does not entail any special additional equipment, has a very short hands-on time (i.e., less than 2 minutes) and has an estimated time of approximately 2 hours from FFPE sample to the final reporting. Since the response time for analyzing KRAS status might be a critical factor, in particular for patients with rapid disease progression, the Idylla^™^ KRAS Mutation Assay could offer new prospects by significantly decreasing turnaround time. Given the fact that the Idylla^™^ KRAS Mutation Assay can be easily implemented in any pathology laboratory setting, even those without prior molecular diagnostics experience, and needs a very short turnaround time to obtain test results, incorporation of the KRAS mutational status in the initial histological report is possible, enabling immediate initiation of targeted therapy. Although the Idylla^™^ KRAS Mutation Assay is not intended to determine all possible KRAS mutations or to quantify allelic frequency, the test is capable of detecting the great majority of the relevant and most prevalent KRAS mutations. However, other techniques should be utilized to obtain quantitative information or complete overviews of mutations present, or to discover new useful tumor biomarkers.

The Idylla^™^ KRAS Mutation Assay is a fully-automated and integrated real-time-PCR-based test with high sensitivity, offering a “one-step” solution for routine application. Up-front sample preparation, apart from possible macro-dissection, is not necessary as FFPE tissue sections are directly loaded into the single-use cartridge. As well, the interpretation of the results is fully automated. Due to the platform characteristics, there is no need for additional molecular infrastructure or a highly skilled staff to perform the test on site. Interestingly, the closure of the leak-proof cartridge after loading the sample limits the risk of cross contamination between different samples. In large molecular laboratories, the Idylla^™^ KRAS Mutation Assay is considered a useful addition to other technologies including NGS, in particular for urgent requests where a very quick turnaround time is needed for occasional patients.

## Conclusion

In conclusion, the Idylla^™^ KRAS Mutation Test on the Idylla^™^ platform is to our knowledge the only fully integrated and automated test for detection of 21 clinically relevant mutations included in current guidelines starting from FFPE tissue samples and represents a relevant solution for simple, highly reliable, and rapid routine determination of the KRAS mutational status needed to guide colorectal cancer therapy.

## Supporting Information

S1 Table Overview of all samples analyzed, with results of Idylla^™^ and of routine reference methods. (DOCX)
