# Supplementary material for: Multi-Center Evaluation of the Fully Automated PCR-Based Idylla™ KRAS Mutation Assay for Rapid KRAS Mutation Status Determination on Formalin-Fixed Paraffin-Embedded Tissue of Human Colorectal Cancer
Source: PLoS One. 2016 Sep 29;11(9):e0163444. doi: 10.1371/journal.pone.0163444 (PMC5042411; doi:10.1371/journal.pone.0163444)
Supplement: S1 Table — (DOCX) [file pone.0163444.s001.docx]

S1 Table: Overview of all samples analyzed, with results of Idylla™ and of routine reference methods.

| **ID** | **Tissue type** | **FFPE tissue section (µm)** | **Number of FFPE tissue sections** | **Tumor cells (%)** | **Tumor area (mm^2^)** | **Macrodissection** | **Idylla™** | **Routine reference method** |
| --- | --- | --- | --- | --- | --- | --- | --- | --- |
| Au_01 | colorectal | 10 | 2 | >50 | 18 | no | G12S | G12S^a^ |
| Au_02 | colorectal | 10 | 2 | >50 | 30 | no | G12V | G12V^a^ |
| Au_03 | colorectal | 10 | 2 | >50 | 60 | no | G12S | G12S^a^ |
| Au_04 | colorectal | 10 | 2 | >50 | 60 | no | G12C | G12C^a^ |
| Au_05 | colorectal | 10 | 2 | >50 | 30 | no | G12D | G12D^a^ |
| Au_06 | colorectal | 10 | 2 | >50 | 30 | no | no mutation | A146T^a^ |
| Au_07 | colorectal | 5 | 1 | >50 | 90 | yes | G12D | G12D^a^ |
| Au_08 | colorectal | 10 | 2 | >50 | 90 | no | G12V | G12V^a^ |
| Au_09 | colorectal | 10 | 2 | >50 | 15 | no | A59T/E/G | A59E^a^ |
| Au_10 | colorectal | 10 | 2 | >30 | 10 | no | Q61K | Q61K^a^ |
| Au_11 | metastasis | 5 | 1 | >50 | 25 | yes | G12D | G12D^a^ |
| Au_12 | colorectal | 10 | 2 | >50 | 45 | no | G12D | G12D^a^ |
| Au_13 | colorectal | 10 | 2 | >50 | 40 | no | G12V | G12D^a^ |
| Au_14 | colorectal | 10 | 2 | >50 | 30 | no | G13D | G13D^a^ |
| Au_15 | metastasis | 5 | 4 | >50 | 5 | yes | G12D | G12D^a^ |
| Au_16 | colorectal | 5 | 4 | >50 | 10 | yes | G12V | G12V^a^ |
| Au_17 | colorectal | 5 | 2 | >50 | 20 | yes | G12V | G12D^a^ |
| Au_18 | metastasis | 5 | 2 | >50 | 15 | yes | no mutation | G12D^a^ |
| Au_19 | colorectal | 5 | 1 | >50 | 20 | yes | G12D | G12D^a^ |
| Au_20 | colorectal | 10 | 2 | >50 | 40 | no | A146P/T/V | A146T^a^ |
| Au_21 | metastasis | 5 | 6 | >30 | 2 | yes | G12A | G12A^a^ |
| Au_23 | colorectal | 5 | 1 | >50 | 50 | yes | G13D | G13D^a^ |
| Au_24 | colorectal | 5 | 2 | >50 | 30 | yes | G12D | G12D^a^ |
| Au_25 | colorectal | 10 | 3 | >30 | 9 | no | G12A | G12A^a^ |
| Au_26 | colorectal | 10 | 1 | >50 | 40 | no | no mutation | no mutation^a^ |
| Au_27 | colorectal | 10 | 5 | >30 | 5 | no | no mutation | no mutation^a^ |
| Au_28 | colorectal | 10 | 5 | >30 | 5 | no | G12S | G12S^a^ |
| Au_29 | colorectal | 10 | 5 | >30 | 2 | no | G12A | G12A^a^ |
| Au_30 | metastasis | 10 | 5 | >30 | 4 | no | no mutation | no mutation^a^ |
| Ba_01 | colorectal | 25 | 1 | >30 | 50 | yes | G12V | G12V^b^ |
| Ba_02 | colorectal | 25 | 1 | >90 | 82 | yes | G12D | G12D^b^ |
| Ba_03 | colorectal | 25 | 1 | >90 | 120 | yes | G13D | G13D^b^ |
| Ba_04 | colorectal | 25 | 1 | >80 | 105 | yes | A146T/V/P | A146T^b,c^ |
| Ba_05 | colorectal | 25 | 1 | >70 | 60 | yes | G13D | G13D^b^ |
| Ba_06 | colorectal | 25 | 1 | >60 | 68 | yes | Q61H | Q61H^b,c^ |
| Ba_07 | colorectal | 25 | 1 | >50 | 15 | yes | G12V | G12V^b^ |
| Ba_08 | colorectal | 25 | 1 | >60 | 36 | yes | G12D | G12D^b^ |
| Ba_09 | liver | 25 | 1 | >70 | 28 | yes | G12R | G12R^b^ |
| Ba_10 | colorectal | 25 | 1 | >50 | 4 | yes | A146T/V/P | A146V^b,c^ |
| Ba_11 | ovary | 25 | 1 | >30 | 60 | yes | G12A | G12A^b^ |
| Ba_12 | colorectal | 25 | 1 | >80 | 60 | yes | A59E/G/T | A59G^b,c^ |
| Ba_13 | colorectal | 25 | 1 | >90 | 78 | yes | A146T/V/P | A146T^b,c^ |
| Ba_14 | colorectal | 16 | 1 | >70 | 11 | no | Q61H | Q61H^b,c^ |
| Ba_15 | colorectal | 25 | 1 | >80 | 72 | yes | no mutation | no mutation^b,c^ |
| Ba_16 | colorectal | 25 | 1 | >60 | 42 | yes | no mutation | no mutation^b,c^ |
| Ba_17 | colorectal | 25 | 1 | >90 | 135 | yes | no mutation | no mutation^b,c^ |
| Ba_18 | colorectal | 25 | 1 | >90 | 49 | yes | no mutation | no mutation^b,c^ |
| Ba_19 | colorectal | 25 | 1 | >60 | 1+2 | yes | no mutation | no mutation^b,c^ |
| Ba_20 | colorectal | 25 | 1 | >60 | 54 | yes | no mutation | no mutation^b,c^ |
| Ba_21 | colorectal | 25 | 1 | >90 | 84 | yes | no mutation | no mutation^b,c^ |
| Ba_22 | colorectal | 25 | 1 | >80 | 100 | yes | no mutation | no mutation^b,c^ |
| Ba_23 | colorectal | 25 | 1 | >60 | 5 | no | no mutation | no mutation^b,c^ |
| Ba_24 | colorectal | 25 | 1 | >80 | 6 | yes | no mutation | no mutation^b,c^ |
| Ba_25 | colorectal | 16 | 1 | >20 | 1 | no | G12V | G12V^b^ |
| Ba_26 | colorectal | 25 | 1 | >60 | 5 | yes | G12D | G12D^b^ |
| Ba_27 | colorectal | 25 | 1 | >80 | 130 | yes | G12V | G12V^b^ |
| Ba_28 | colorectal | 25 | 1 | >30 | 10 | yes | G13D | G13D^b^ |
| Ba_29 | colorectal | 25 | 1 | >20 | 8 | yes | G12V | G12V^b^ |
| Ba_30 | colorectal | 16 | 1 | >100 | 14 | no | K117N | K117N^b,c^ |
| Ba_31 | colorectal | 10 | 4 | ND | 14 | no | G12S | invalid^b^ |
| Bi_01 | liver | 6 | 3 | 70 | 12 | no | no mutation | G13C^b,d^ |
| Bi_02 | colon | 6 | 1 | 40 | 300 | no | Q61L/R | Q61L^c,d^ |
| Bi_03 | rectum | 6 | 1 | 30 | 450 | no | no mutation | no mutation^b,c,d^ |
| Bi_04 | colon | 6 | 1 | 20 | 398 | no | G12C | no mutation^b,c,d^ |
| Bi_05 | colon | 6 | 1 | 40 | 192 | no | no mutation | no mutation^b,c,d^ |
| Bi_06 | liver | 6 | 1 | 70 | 450 | no | A146P/T/V | A146T^c^ |
| Bi_07 | rectum | 6 | 4 | 30 | 60 | no | no mutation | G13C^b^ |
| Bi_08 | colon | 6 | 1 | 30 | 300 | no | A146P/T/V | A146V^c^ |
| Bi_09 | colon | 6 | 4 | 70 | 44.8 | no | no mutation | no mutation^b,c,d^ |
| Bi_10 | colon | 6 | 4 | 30 | 24.5 | no | no mutation | no mutation^b,c,d^ |
| Bi_11 | colon | 6 | 1 | 30 | 300 | no | G12A | G12A^b,d^ |
| Bi_12 | rectum | 6 | 3 | 10 | 50 | no | G12S | G12S^b,d^ |
| Bi_13 | colon | 6 | 3 | 60 | 30 | no | G12D | G12D^b,d^ |
| Bi_14 | rectum | 6 | 1 | 60 | 200 | no | Q61H | Q61H^c,d^ |
| Bi_15 | liver | 6 | 1 | 10 | 340 | no | A146P/T/V | no mutation^b,c,d^ |
| Bi_16 | rectal sigmoid | 6 | 1 | 10 | 480 | no | no mutation | no mutation^b,c,d^ |
| Bi_17 | rectum | 6 | 3 | 40 | 30 | no | G12C | G12C^b,d^ |
| Bi_19 | colon | 6 | 4 | 30 | 17 | no | G13D | G13D^b,d^ |
| Bi_20 | liver | 6 | 1 | 10 | 378 | no | G12V | G12V^b,d^ |
| Bi_21 | liver | 6 | 1 | 50 | 378 | no | G12V | G12V^b,d^ |
| Bi_22 | coloc | 6 | 1 | 20 | 480 | no | no mutation | no mutation^b,c,d^ |
| Bi_23 | liver | 6 | 1 | 20 | 360 | no | no mutation | no mutation^b,c^ |
| Bi_24 | anal margin | 6 | 1 | 20 | 360 | no | no mutation | no mutation^b,c^ |
| Bi_25 | liver | 6 | 1 | 10 | 202.5 | no | no mutation | no mutation^b,c^ |
| Bi_26 | colon | 6 | 4 | 40 | 17.5 | no | G13D | G13D^b^ |
| Bi_27 | colon | 6 | 3 | 60 | 21 | no | G12R | G12R^b,d^ |
| Bi_28 | colon | 6 | 1 | 20 | 512 | no | no mutation | no mutation^b,c^ |
| Bi_29 | colon | 6 | 1 | 20 | 480 | no | no mutation | no mutation^b,c^ |
| Bi_30 | liver | 6 | 4 | 20 | 20 | no | no mutation | no mutation^b,c^ |
| Bi_31 | colon | 6 | 4 | 20 | 70 | no | A146P/T/V | A146T^c^ |
| Bi_32 | liver | 6 | 1 | 20 | 320 | no | G13D | G13D^b^ |
| Bi_33 | liver | 6 | 1 | 20 | 342 | no | no mutation | no mutation^b,c^ |
| Br_01 | colorectal | 10 | 4 | 10 | 25 | yes | G12C | G12C^e^ |
| Br_02 | colorectal | 10 | 1 | 40 | 300 | no | no mutation | no mutation^e^ |
| Br_03 | colorectal | 10 | 2 | 30 | 25 | yes | no mutation | no mutation^e^ |
| Br_04 | colorectal | 10 | 1 | 20 | 250 | no | no mutation | no mutation^e^ |
| Br_05 | colorectal | 10 | 1 | 30 | 75 | no | no mutation | no mutation^e^ |
| Br_06 | colorectal | 10 | 2 | 25 | 150 | yes | A146T/V/P | A146T^e^ |
| Br_07 | colorectal | 10 | 4 | 30 | 50 | yes | G12V | G12V^e^ |
| Br_08 | colorectal | 10 | 1 | 60 | 150 | yes | no mutation | no mutation^e^ |
| Br_09 | colorectal | 10 | 1 | 60 | 200 | no | G12C | G12C^e^ |
| Br_10 | colorectal | 10 | 12 | 10 | 10 | yes | no mutation | no mutation^e^ |
| Br_11 | colorectal | 10 | 2 | 50 | 25 | yes | A146T/V/P | A146T^e^ |
| Br_12 | colorectal | 10 | 4 | 10 | 25 | no | no mutation | no mutation^e^ |
| Br_13 | colorectal | 10 | 1 | 40 | 100 | yes | G13D | G13D^e^ |
| Br_14 | colorectal | 10 | 6 | 30 | 10 | no | no mutation | no mutation^e^ |
| Br_15 | colorectal | 10 | 2 | 20 | 75 | yes | no mutation | no mutation^e^ |
| Br_16 | colorectal | 10 | 1 | 50 | 150 | yes | A59E/G/T | A59T ^e^ |
| Br_17 | colorectal | 10 | 2 | 50 | 25 | yes | A59E/G/T | A59E^e^ |
| Br_18 | colorectal | 10 | 1 | 50 | 150 | yes | G12S | G12S^e^ |
| Br_19 | colorectal | 10 | 2 | 60 | 25 | yes | Q61R/L | Q61R^e^ |
| Br_20 | colorectal | 10 | 2 | 30 | 100 | yes | G12V | G12V^e^ |
| Br_21 | colorectal | 10 | 1 | 50 | 200 | yes | K117N | K117N^e^ |
| Br_22 | colorectal | 10 | 3 | 20 | 75 | yes | no mutation | no mutation^e^ |
| Br_23 | colorectal | 10 | 2 | 50 | 100 | yes | G12D | G12D^e^ |
| Br_24 | colorectal | 10 | 3 | 30 | 25 | no | no mutation | no mutation^e^ |
| Br_25 | colorectal | 10 | 1 | 30 | 350 | no | G13D | G13D^e^ |
| Br_26 | colorectal | 10 | 1 | 60 | 125 | yes | no mutation | no mutation^e^ |
| Br_27 | colorectal | 10 | 3 | 20 | 25 | yes | no mutation | no mutation^e^ |
| Br_28 | colorectal | 10 | 6 | 10 | 25 | yes | G12V | G12V^e^ |
| Br_29 | colorectal | 10 | 2 | 50 | 50 | yes | G12A | G12A^e^ |
| Br_30 | colorectal | 10 | 2 | 30 | 100 | no | G12D | G12D^e^ |
| Co_01 | colorectal | 5 | 2 | 40 | 15 | no | G12V | G12V^e^ |
| Co_02 | lung | 5 | 2 | 25 | 100 | no | A146P/T/V | A146T^e^ |
| Co_03 | colorectal | 5 | 2 | ND | ND | no | G12D | G12D^e^ |
| Co_04 | colorectal | 5 | 2 | ND | ND | no | G13D | G13D^e^ |
| Co_05 | colorectal | 5 | 2 | 30 | 10 | no | no mutation | no mutation^e^ |
| Co_06 | colorectal | 5 | 2 | 60 | 150 | no | no mutation | no mutation^e^ |
| Co_07 | colorectal | 5 | 2 | 95 | 300 | no | G12A | codon 12/13^d^ |
| Co_08 | colorectal | 5 | 2 | 90 | 300 | no | no mutation | no mutation^e^ |
| Co_09 | colorectal | 5 | 2 | 70 | 100 | no | no mutation | no mutation^e^ |
| Co_10 | colorectal | 5 | 2 | 10 | 10 | no | no mutation | no mutation^e^ |
| Co_11 | colorectal | 5 | 2 | 60 | 45 | no | G12D | codon 12/13^d^ |
| Co_12 | colorectal | 5 | 2 | 40 | 10 | no | G13D | codon 12/13^d^ |
| Co_13 | colorectal | 5 | 2 | 30 | 50 | no | no mutation | codon 12/13^d^ |
| Co_14 | colorectal | 5 | 2 | ND | ND | no | no mutation | no mutation^e^ |
| Co_15 | colorectal | 5 | 2 | ND | ND | no | no mutation | no mutation^e^ |
| Co_16 | colorectal | 5 | 2 | 160 | 100 | no | G12D | G12D^e^ |
| Co_17 | colorectal | 5 | 2 | ND | ND | no | G12D | codon 12/13^d^ |
| Co_18 | colorectal | 5 | 2 | ND | ND | no | no mutation | no mutation^e^ |
| Co_19 | colorectal | 5 | 2 | 80 | 50 | no | G12D | codon 12/13^d^ |
| Co_20 | colorectal | 5 | 2 | 80 | ND | no | no mutation | no mutation^e^ |
| Co_21 | colorectal | 5 | 2 | 50 | 50 | no | G12C | G12C^e^ |
| Co_22 | colorectal | 5 | 2 | 40 | 10 | no | no mutation | no mutation^e^ |
| Co_23 | colorectal | 5 | 2 | 25 | 100 | no | no mutation | no mutation^e^ |
| Co_24 | colorectal | 5 | 2 | 60 | 25 | no | no mutation | no mutation^e^ |
| Co_25 | colorectal | 5 | 2 | 15 | 10 | no | G12V | codon 12/13^d^ |
| Co_26 | colorectal | 5 | 2 | 80 | 50 | no | no mutation | no mutation^e^ |
| Co_27 | colorectal | 5 | 2 | 40 | 75 | no | no mutation | no mutation^e^ |
| Co_28 | colorectal | 5 | 2 | ND | ND | no | no mutation | no mutation^e^ |
| Co_29 | colorectal | 5 | 2 | 135 | 10 | no | G13D | G13D^e^ |
| Du_01 | colorectal | 10 | 1 | 30 | 450 | no | no mutation | no mutation^d^ |
| Du_02 | colorectal | 10 | 1 | 40 | 400 | no | no mutation | no mutation^d^ |
| Du_03 | colorectal | 10 | 1 | 30 | 60 | no | no mutation | no mutation^d^ |
| Du_04 | colorectal | 10 | 1 | 80 | 30 | no | G12D | codon 12/13^d^ |
| Du_05 | colorectal | 10 | 1 | 40 | 100 | no | A146P/T/V | no mutation^d^ |
| Du_06 | colorectal | 10 | 1 | 30 | 80 | no | no mutation | no mutation^d^ |
| Du_07 | colorectal | 10 | 1 | 40 | 50 | no | G12D | codon 12/13^d^ |
| Du_08 | colorectal | 10 | 1 | 50 | 20 | no | no mutation | no mutation^d^ |
| Du_09 | colorectal | 10 | 1 | 60 | 80 | no | G12V | codon 12/13^d^ |
| Du_10 | colorectal | 10 | 1 | 30 | 80 | no | G12D | codon 12/13^d^ |
| Du_11 | colorectal | 10 | 1 | 10 | 10 | no | no mutation | no mutation^d^ |
| Du_12 | colorectal | 10 | 1 | 40 | 50 | no | G12D | codon 12/13^d^ |
| Du_13 | colorectal | 10 | 1 | 40 | 600 | no | no mutation | no mutation^d^ |
| Du_14 | colorectal | 10 | 1 | 30 | 100 | no | G13D | codon 12/13^d^ |
| Du_15 | colorectal | 10 | 1 | 30 | 100 | no | no mutation | no mutation^d^ |
| Du_16 | colorectal | 10 | 1 | 30 | 100 | no | no mutation | no mutation^d^ |
| Du_17 | colorectal | 10 | 1 | 70 | 80 | no | G12V | codon 12/13^d^ |
| Du_18 | colorectal | 10 | 1 | 40 | 600 | no | no mutation | no mutation^d^ |
| Du_19 | colorectal | 10 | 1 | 80 | 600 | no | G12D | codon 12/13^d^ |
| Du_20 | colorectal | 10 | 1 | 50 | 400 | no | G12C | codon 12/13^d^ |
| Du_21 | colorectal | 10 | 1 | 50 | 150 | no | no mutation | no mutation^d^ |
| Du_22 | colorectal | 10 | 1 | 60 | 300 | no | G12V | codon 12/13^d^ |
| Du_23 | colorectal | 10 | 1 | 40 | 300 | no | no mutation | no mutation^d^ |
| Du_24 | colorectal | 10 | 1 | 10 | 100 | no | G13D | codon 12/13^d^ |
| Du_25 | colorectal | 10 | 1 | 40 | 100 | no | G12V | codon 12/13^d^ |
| Du_26 | colorectal | 10 | 1 | 50 | 63 | no | no mutation | codon 12/13^d^ |
| Du_27 | colorectal | 10 | 1 | 50 | 50 | no | no mutation | no mutation^d^ |
| Du_28 | colorectal | 10 | 1 | 20 | 60 | no | G12D | codon 12/13^d^ |
| Du_29 | colorectal | 10 | 1 | 70 | 60 | no | Q61L/R | codon 61^d^ |
| Du_30 | colorectal | 10 | 1 | 20 | 60 | no | G13D | codon 12/13^d^ |
| Du_31 | colorectal | 10 | 1 | 40 | 60 | no | G12D | codon 12/13^d^ |
| Du_32 | colorectal | 10 | 1 | 50 | 400 | no | no mutation | no mutation^d^ |
| Lo_01 | colon | 5 | 1 | 40 | 250 | no | no mutation | no mutation^d^ |
| Lo_02 | colon | 5 | 1 | 50 | 300 | no | no mutation | no mutation^d^ |
| Lo_03 | colon | 5 | 1 | 60 | 150 | no | no mutation | no mutation^d^ |
| Lo_04 | colon | 5 | 1 | 60 | 500 | no | G12C | codon 12/13^d^ |
| Lo_05 | colon | 5 | 1 | 60 | 300 | no | G12D | codon 12/13^d^ |
| Lo_06 | colon | 5 | 1 | 70 | 500 | no | no mutation | no mutation^d^ |
| Lo_07 | colon | 5 | 1 | 40 | 340 | no | no mutation | no mutation^d^ |
| Lo_08 | colon | 5 | 1 | 55 | 300 | no | G12C | codon 12/13^d^ |
| Lo_09 | colon | 5 | 1 | 70 | 550 | no | G13D | codon 12/13^d^ |
| Lo_10 | colon | 5 | 1 | 70 | 200 | no | G12D | codon 12/13^d^ |
| Lo_11 | omentum | 5 | 1 | 15 | 160 | no | G13D | codon 12/13^d^ |
| Lo_12 | colon | 5 | 1 | 90 | 250 | no | A146P/T/V | no mutation^d^ |
| Lo_13 | colon | 5 | 1 | 50 | 400 | no | no mutation | no mutation^d^ |
| Lo_14 | colon | 5 | 1 | 40 | 360 | no | G12D | codon 12/13^d^ |
| Lo_15 | colon | 5 | 1 | 30 | 300 | no | no mutation | no mutation^d^ |
| Lo_16 | small bowel | 5 | 1 | 80 | 300 | no | G13D | codon 12/13^d^ |
| Lo_17 | colon | 5 | 1 | 20 | 375 | no | no mutation | no mutation^d^ |
| Lo_18 | vagina | 5 | 1 | 50 | 230 | no | no mutation | no mutation^d^ |
| Lo_19 | colon | 5 | 4 | 20 | 60 | no | G12C | codon 12/13^d^ |
| Lo_20 | rectum | 5 | 1 | 95 | 200 | no | G12C | codon 12/13^d^ |
| Lo_21 | colon | 5 | 1 | 95 | 330 | no | no mutation | no mutation^d^ |
| Lo_22 | rectum | 5 | 2 | 30 | 60 | no | G13D | codon 12/13^d^ |
| Lo_23 | colon | 5 | 1 | 65 | 500 | no | no mutation | codon 12/13^d^ |
| Lo_24 | colon | 5 | 1 | 70 | 375 | no | G12D | codon 12/13^d^ |
| Lo_25 | colon, hepatic flexure | 5 | 1 | 30 | 60 | no | G12D | codon 12/13^d^ |
| Lo_26 | colon | 5 | 1 | 70 | 50 | no | G12V | codon 12/13^d^ |
| Lo_27 | cecum | 5 | 1 | 60 | 50 | no | G12C | codon 12/13^d^ |
| Lo_28 | colon, splenic flexure | 5 | 2 | 46 | 50 | no | G12A | codon 12/13^d^ |
| Lo_29 | cecum | 5 | 2 | 40 | 70 | no | G13D | codon 12/13^d^ |
| Lo_30 | colon | 5 | 1 | 70 | 500 | no | G13D | codon 12/13^d^ |
| Lo_31 | colon | 5 | 2 | 40 | 50 | no | no mutation | no mutation^d^ |
| Mi_01 | liver | 10 | 1 | 60 | 200 | no | G12V | G12V^a,f^ |
| Mi_02 | colon | 10 | 1 | 70 | 200 | no | G13D | G13D^a,f^ |
| Mi_03 | lymph node | 5 | 2 | 60 | 70 | no | Q61H | no mutation^a,f^ |
| Mi_04 | colon | 10 | 1 | 90 | 225 | no | no mutation | no mutation^a,f^ |
| Mi_05 | ovary | 5 | 2 | 70 | 300 | no | A146T/V/P | A146T^a,f^ |
| Mi_06 | liver | 5 | 3 | 50 | 15 | yes | G13D | G13D^a,f^ |
| Mi_07 | colon | 10 | 1 | 60 | 125 | no | Q61H | no mutation^a,f^ |
| Mi_08 | colon | 5 | 3 | 70 | 15 | no | no mutation | no mutation^a,f^ |
| Mi_09 | colon | 5 | 2 | 20 | 90 | no | G12D | G12D^a,f^ |
| Mi_10 | liver | 5 | 2 | 60 | 50 | no | Q61H | no mutation^a,f^ |
| Mi_11 | colon | 10 | 1 | 80 | 200 | no | G12D | G12D^a,f^ |
| Mi_12 | brain | 5 | 2 | 70 | 160 | no | G12D | G12D^a,f^ |
| Mi_13 | colon | 10 | 1 | 50 | 225 | no | G12D | no mutation^a,f^ |
| Mi_14 | colon | 10 | 1 | 70 | 260 | no | no mutation | no mutation^a,f^ |
| Mi_15 | colon | 10 | 1 | 70 | 260 | no | no mutation | no mutation^a,f^ |
| Mi_16 | colon | 10 | 1 | 80 | 170 | no | G13D | G13D^a,f^ |
| Mi_17 | lung | 5 | 2 | 50 | 45 | no | A146T/V/P | A146P^a,f^ |
| Mi_18 | rectum | 10 | 1 | 95 | 250 | no | no mutation | no mutation^a,f^ |
| Mi_19 | rectum | 10 | 1 | 80 | 130 | no | no mutation | no mutation^a,f^ |
| Mi_20 | colon | 5 | 2 | 80 | 25 | no | G13D | G13D^a,f^ |
| Mi_21 | colon | 10 | 1 | 80 | 150 | no | G12D | G12D^a,f^ |
| Mi_22 | colon | 5 | 3 | 20 | 75 | yes | G12A | G12A^a,f^ |
| Mi_23 | rectum | 10 | 1 | 80 | 400 | no | G12V | G12V^a,f^ |
| Mi_24 | colon | 10 | 1 | 50 | 110 | no | A59T | A59T^a,f^ |
| Mi_25 | sigma | 10 | 1 | 80 | 220 | no | G12C | G12C^a,f^ |
| Mi_26 | liver | 10 | 1 | 80 | 300 | no | Q61H | Q61H^a,f^ |
| Mi_27 | rectum | 10 | 1 | 70 | 200 | no | G12S | G12S^a,f^ |
| Mi_28 | colon | 10 | 1 | 70 | 140 | no | K117N | K117N^a,f^ |
| Mi_29 | liver | 10 | 1 | 60 | 290 | no | G12R | G12R^a,f^ |
| Mi_30 | colon | 5 | 3 | 40 | 60 | no | Q61R | Q61R^a,f^ |
| Mi_31 | colon | 10 | 1 | 80 | 325 | no | Q61H | no mutation^a,f^ |
| Mi_32 | rectum | 5 | 3 | 90 | 15 | no | G12V | G12V^a,f^ |
| Mi_33 | colon | 10 | 1 | 60 | 40 | no | G12D | G12D^a,f^ |
| Mi_34 | liver | 5 | 3 | 80 | 25 | no | Q61H | G12V^a,f^ |
| Mo_01 | colorectal | 10 | 10 | 80 | 120 | yes | A146P/T/V | A146T^f,g^ |
| Mo_02 | colorectal | 10 | 10 | 70 | 91 | yes | G13D | G13D^f,g^ |
| Mo_03 | colorectal | 10 | 10 | 80 | 300 | yes | G13D | G13D^f,g^ |
| Mo_04 | colorectal | 10 | 10 | 60 | 400 | yes | G12V | G12V^f,g^ |
| Mo_05 | colorectal | 10 | 10 | 70 | 130 | yes | G12S | G12S^f,g^ |
| Mo_06 | colorectal | 10 | 10 | 70 | 50 | yes | G12V | G12V^f,g^ |
| Mo_07 | colorectal | 10 | 10 | 60 | 225 | yes | G13D | G13D^f,g^ |
| Mo_08 | colorectal | 10 | 10 | 70 | 640 | yes | A146P/T/V | A146T^f,g^ |
| Mo_09 | colorectal | 10 | 10 | 70 | 56 | yes | G12D | G12D^f,g^ |
| Mo_10 | colorectal | 10 | 10 | 30 | 400 | yes | G12D | G12D^f,g^ |
| Mo_11 | colorectal | 10 | 10 | 80 | 121 | yes | G12C | G12C^f,g^ |
| Mo_12 | colorectal | 10 | 10 | 70 | 70 | yes | G12C | G12C^f,g^ |
| Mo_13 | colorectal | 10 | 10 | 60 | 170 | yes | G12R | G12R^f,g^ |
| Mo_14 | colorectal | 10 | 10 | 60 | 65 | yes | no mutation | G13D^a,f,g^ |
| Mo_15 | colorectal | 10 | 10 | 60 | 165 | yes | G12V | G12V^f,g^ |
| Mo_16 | colorectal | 10 | 10 | 70 | 225 | yes | no mutation | no mutation^f,g^ |
| Mo_17 | colorectal | 10 | 10 | 60 | 110 | yes | no mutation | no mutation^f,g^ |
| Mo_18 | colorectal | 10 | 10 | 90 | 120 | yes | no mutation | no mutation^f,g^ |
| Mo_19 | colorectal | 10 | 10 | 80 | 306 | yes | no mutation | no mutation^f,g^ |
| Mo_20 | colorectal | 10 | 10 | 50 | 75 | yes | no mutation | no mutation^f,g^ |
| Mo_21 | colorectal | 10 | 10 | 40 | 99 | yes | no mutation | no mutation^f,g^ |
| Mo_22 | colorectal | 10 | 10 | 60 | 55 | yes | no mutation | no mutation^f,g^ |
| Mo_23 | colorectal | 10 | 10 | 70 | 56 | yes | no mutation | no mutation^f,g^ |
| Mo_24 | colorectal | 10 | 10 | 80 | 121 | yes | no mutation | no mutation^f,g^ |
| Mo_25 | colorectal | 10 | 10 | 70 | 391 | yes | no mutation | no mutation^f,g^ |
| Mo_26 | colorectal | 10 | 10 | 70 | 50 | yes | no mutation | no mutation^f,g^ |
| Mo_27 | colorectal | 10 | 10 | 60 | 216 | yes | no mutation | no mutation^f,g^ |
| Mo_28 | colorectal | 10 | 10 | 70 | 221 | yes | no mutation | no mutation^f,g^ |
| Mo_29 | colorectal | 10 | 10 | 80 | 510 | yes | no mutation | no mutation^f,g^ |
| Mo_30 | colorectal | 10 | 10 | 30 | 374 | yes | no mutation | no mutation^f,g^ |
| Mo_31 | colorectal | 10 | 10 | 50 | 140 | yes | no mutation | G12F^a,f,g^ |
| Mo_32 | colorectal | 10 | 10 | 10 | 30 | yes | no mutation | G12-G13>AR^a,f,g^ |
| Ox_01 | colorectal | 5 | 1 | >50 | 50-600 | yes | G12D | G12D^d^ |
| Ox_02 | colorectal | 5 | 1 | >50 | 50-600 | yes | no mutation | no mutation^d^ |
| Ox_03 | colorectal | 5 | 1 | >50 | 50-600 | yes | G12V | G12V^d^ |
| Ox_04 | colorectal | 5 | 1 | >50 | 50-600 | yes | G12V | G12V^d^ |
| Ox_05 | colorectal | 5 | 1 | >50 | 50-600 | yes | no mutation | no mutation^d^ |
| Ox_06 | colorectal | 5 | 1 | >50 | 50-600 | yes | no mutation | no mutation^d^ |
| Ox_07 | colorectal | 5 | 1 | >50 | 50-600 | yes | no mutation | no mutation^d^ |
| Ox_08 | colorectal | 5 | 1 | >50 | 50-600 | yes | G12A | G12A^d^ |
| Ox_09 | colorectal | 5 | 1 | >50 | 50-600 | yes | no mutation | no mutation^d^ |
| Ox_10 | colorectal | 5 | 1 | >50 | 50-600 | yes | G12D | G12D^d^ |
| Ox_11 | colorectal | 5 | 1 | >50 | 50-600 | yes | no mutation | no mutation^d^ |
| Ox_12 | colorectal | 5 | 1 | >50 | 50-600 | yes | no mutation | no mutation^d^ |
| Ox_13 | colorectal | 5 | 1 | >50 | 50-600 | yes | G12V | no mutation^d^ |
| Ox_14 | colorectal | 5 | 1 | >50 | 50-600 | yes | no mutation | no mutation^d^ |
| Ox_15 | colorectal | 5 | 1 | >50 | 50-600 | yes | G12D | G12D^d^ |
| Ox_16 | colorectal | 5 | 1 | >50 | 50-600 | yes | no mutation | no mutation^d^ |
| Ox_17 | colorectal | 5 | 1 | >50 | 50-600 | yes | no mutation | no mutation^d^ |
| Ox_18 | colorectal | 5 | 1 | >50 | 50-600 | yes | G12C | G12C^d^ |
| Ox_19 | colorectal | 5 | 1 | >50 | 50-600 | yes | G12R | G12R^d^ |
| Ox_20 | colorectal | 5 | 1 | >50 | 50-600 | yes | G12D | G12D^d^ |
| Ox_21 | colorectal | 5 | 1 | >50 | 50-600 | yes | no mutation | no mutation^d^ |
| Ox_22 | colorectal | 5 | 1 | >50 | 50-600 | yes | G13D | G13D^d^ |
| Ox_23 | colorectal | 5 | 1 | >50 | 50-600 | yes | no mutation | no mutation^d^ |
| Ox_24 | colorectal | 5 | 1 | >50 | 50-600 | yes | no mutation | no mutation^d^ |
| Ox_25 | colorectal | 5 | 1 | >50 | 50-600 | yes | G12D | G12D^d^ |
| Ox_26 | colorectal | 5 | 1 | >50 | 50-600 | yes | G12D | G12D^d^ |
| Ox_27 | colorectal | 5 | 1 | >50 | 50-600 | yes | Q61L/R | Q61L/R^d^ |
| Ox_28 | colorectal | 5 | 1 | >50 | 50-600 | yes | Q61L/R | no mutation^d^ |
| Ox_29 | colorectal | 5 | 1 | >50 | 50-600 | yes | G13D | G13D^d^ |
| Ox_30 | colorectal | 5 | 1 | >50 | 50-600 | yes | G13D | G13D^d^ |
| Pa_01 | colorectal | 10 | 1 | 90 | 90 | no | G12D | G12D^a^ |
| Pa_02 | liver | 10 | 6 | 35 | 8.5 | no | G13D | G13D^a^ |
| Pa_03 | liver | 10 | 1 | 75 | 240 | no | G12C | G12C^a^ |
| Pa_04 | colorectal | 10 | 1 | 65 | 168 | no | A146T/V/P | A146V^a^ |
| Pa_05 | colorectal | 10 | 3 | 80 | 20 | no | G12A | G12A^a^ |
| Pa_06 | liver | 10 | 1 | 80 | 234 | no | G12A | G12A^a^ |
| Pa_07 | colorectal | 10 | 5 | 15 | 11 | no | A146T/V/P | A146T^a^ |
| Pa_08 | colorectal | 10 | 1 | 50 | 190 | no | no mutation | no mutation^a^ |
| Pa_09 | colorectal | 10 | 1 | 70 | 209 | no | G12D | G12D^a^ |
| Pa_10 | colorectal | 10 | 1 | 60 | 145 | no | no mutation | no mutation^a^ |
| Pa_11 | colorectal | 10 | 1 | 70 | 182 | no | no mutation | no mutation^a^ |
| Pa_12 | liver | 10 | 1 | 40 | 70 | no | Q61R/L | no mutation^a^ |
| Pa_13 | colorectal | 10 | 1 | 65 | 198 | no | G13D | G13D^a^ |
| Pa_14 | lung | 10 | 1 | 70 | 105 | no | G12V | G12V^a^ |
| Pa_15 | colorectal | 10 | 1 | 45 | 70 | no | G12A | G12A^a^ |
| Pa_16 | liver | 10 | 1 | 65 | 117 | no | G12D | G12D^a^ |
| Pa_17 | colorectal | 10 | 1 | 40 | 64 | no | G12V | G12V^a^ |
| Pa_18 | colorectal | 10 | 1 | 70 | 192 | no | G12D | G12D^a^ |
| Pa_19 | liver | 10 | 1 | 70 | 180 | no | G12D | G12D^a^ |
| Pa_20 | colorectal | 10 | 7 | 45 | 7.5 | no | G12V | G12V^a^ |
| Pa_21 | colorectal | 10 | 1 | 55 | 78 | no | G12C | G12C^a^ |
| Pa_22 | liver | 10 | 2 | 70 | 30 | no | G12S | G12S^a^ |
| Pa_23 | colorectal | 10 | 5 | 40 | 12 | no | G13D | G13D^a^ |
| Pa_24 | liver | 10 | 1 | 30 | 55 | no | G12S | G12S^a^ |
| Pa_25 | colorectal | 10 | 1 | 45 | 72 | no | G12V | G12V^a^ |
| Pa_26 | colorectal | 10 | 1 | 70 | 126 | no | G12D | G12D^a^ |
| Pa_27 | colorectal | 10 | 1 | 40 | 120 | no | G12D | G12D^a^ |
| Pa_28 | colorectal | 10 | 1 | 35 | 94 | no | no mutation | G13R^a^ |
| Pa_29 | colorectal | 10 | 1 | 50 | 90 | no | G12V | G12V^a^ |
| Pa_30 | colorectal | 10 | 1 | 60 | 78 | no | G13D | G13D^a^ |
| To_01 | colorectal | 10 | 1 | 60 | 621 | no | G12D | G12A+G12D^b,f^ |
| To_02 | colorectal | 3 | 1 | 30 | 45 | yes | A146T/V/P | A146T^c,f^ |
| To_03 | colorectal | 10 | 1 | 65 | 400 | no | Q61R/L | Q61R^c,f^ |
| To_04 | colorectal | 10 | 1 | 35 | 600 | no | invalid | G13D^b,f^ |
| To_05 | colorectal | 3 | 1 | 50 | 60 | yes | G12D | G12D^b,f^ |
| To_06 | adrenal gland | 10 | 1 | 50 | 506 | no | no mutation | no mutation^b,c,f^ |
| To_07 | colorectal | 10 | 1 | 60 | 220 | no | no mutation | no mutation^b,c,f^ |
| To_08 | colorectal | 3 | 1 | 50 | 232 | no | no mutation | no mutation^b,c,f^ |
| To_09 | colorectal | 3 | 8 | 80 | 64 | yes | no mutation | no mutation^b,c,f^ |
| To_10 | colorectal | 3 | 1 | 50 | 159 | yes | G13D | G13D^b,f^ |
| To_11 | colorectal | 3 | 1 | 80 | 52 | yes | G12V | G12V^b,f^ |
| To_12 | colorectal | 3 | 1 | 70 | 130 | yes | Q61H | Q61H^c,f^ |
| To_13 | colorectal | 3 | 1 | 60 | 70 | yes | G12V | G12V^b,f^ |
| To_14 | colorectal | 3 | 1 | 60 | 234 | yes | A146T/V/P | A146V^c,f^ |
| To_15 | colorectal | 3 | 8 | 60 | 64 | no | Q61H | Q61H^c,f^ |
| To_16 | colorectal | 3 | 1 | 30 | 60 | yes | G12D | G12D^b,f^ |
| To_17 | colorectal | 3 | 1 | 40 | 100 | yes | G12V | G12V^b,f^ |
| To_18 | colorectal | 3 | 1 | 70 | 112 | yes | Q61K | G60G+Q61K^c,f^ |
| To_19 | colorectal | 3 | 1 | 60 | 91 | yes | no mutation | G13C^b,f^ |
| To_20 | liver | 3 | 2 | 45 | 46 | no | G13D | G13D^b,f^ |
| To_21 | colorectal | 3 | 1 | 50 | 200 | yes | no mutation | no mutation^b,c,f^ |
| To_22 | colorectal | 3 | 1 | 40 | 63 | yes | no mutation | no mutation^b,c,f^ |
| To_23 | colorectal | 3 | 10 | 35 | 50 | yes | no mutation | no mutation^b,c,f^ |
| To_24 | colorectal | 3 | 1 | 60 | 121 | yes | no mutation | no mutation^b,c,f^ |
| To_25 | colorectal | 10 | 2 | 85 | 400 | no | K117N | K117N^c,f^ |
| To_26 | colorectal | 3 | 2 | 35 | 23 | no | G12D | G12D^b,f^ |
| To_27 | colorectal | 3 | 2 | 15 | 20 | no | no mutation | no mutation^b,c,f^ |
| To_28 | colorectal | 3 | 1 | 30 | 10 | yes | no mutation | no mutation^b,c,f^ |
| To_29 | colorectal | 10 | 3 | 30 | 300 | no | invalid | G12V^b,f^ |
| To_30 | colorectal | 10 | 3 | 40 | 990 | no | G12D | G12D^b,f^ |
| To_31 | colorectal | 10 | 1 | 30 | 442 | no | no mutation | no mutation^b,c,f^ |
| To_32 | colorectal | 3 | 1 | 40 | 60 | yes | no mutation | no mutation^b,c,f^ |
| To_33 | colorectal | 3 | 1 | 70 | 125 | yes | no mutation | no mutation^b,c,f^ |
| To_34 | colorectal | 3 | 1 | 70 | 273 | yes | G12D | G12D^b,f^ |

ND, not determined.

^a^ Sanger sequencing.

^b^ therascreen^®^ KRAS Pyro Kit (Qiagen).

^c^ therascreen^®^ RAS Extension Pyro Kit (Qiagen).

^d^ cobas^®^ KRAS Mutation Test (Roche).

^e^ Ion Torrent AmpliSeq™ Colon and Lung Cancer Research Panel (Life Technologies).

^f^ HRM screening.

^g^ In-house pyrosequencing.
